# Bilateral macular hemorrhage in a patient with COVID-19

**DOI:** 10.1016/j.ajoc.2020.100958

**Published:** 2020-10-09

**Authors:** Rossella D'Aloisio, Vincenzo Nasillo, Matteo Gironi, Rodolfo Mastropasqua

**Affiliations:** a Ophthalmology Clinic, Department of Medicine and Science of Ageing, University G. D'Annunzio Chieti-Pescara, Chieti, Italy; b Section of Hematology, University of Modena and Reggio Emilia, Azienda Ospedaliero-Universitaria di Modena, Policlinico, Italy; c Ophthalmology Clinic, University of Modena and Reggio Emilia, Azienda Ospedaliero-Universitaria di Modena, Policlinico, Italy

**Keywords:** Macular hemorrhage, SARS-CoV-2, Hereditary spherocytosis

## Abstract

**Purpose:**

We report a case of a patient with a known hereditary spherocytosis who developed a bilateral macular hemorrhage in concurrence with severe acute respiratory syndrome coronavirus 2 (SARS-CoV-2)-related respiratory syndrome.

**Observations:**

Blood tests showed severe hemolytic anemia. Interestingly, the peripheral blood smear demonstrated a mixed pathogenesis of the hemolytic process (cold-agglutinin-mediated and non-immune-mediated due to spherocytosis).

**Conclusions and Importance:**

We argue that SARS-CoV-2 could have triggered the hemolytic process, which led to retinal hemorrhages due to endothelial anoxia from the low oxygen carrying capacity.

## 1 Introduction

Coronavirus disease 2019 (COVID-19) has spread rapidly across the globe to cause a pandemic. Coronavirus has been previously reported to be associated with conjunctivitis in humans^1^ whereas retinal disorders have been described in experimental animal models. We present an unusual case in which a bilateral macular hemorrhage occurred concurrently with the onset of severe SARS-CoV-2 respiratory syndrome.

## 2 Case report/case presentation

**Fig. 1:**
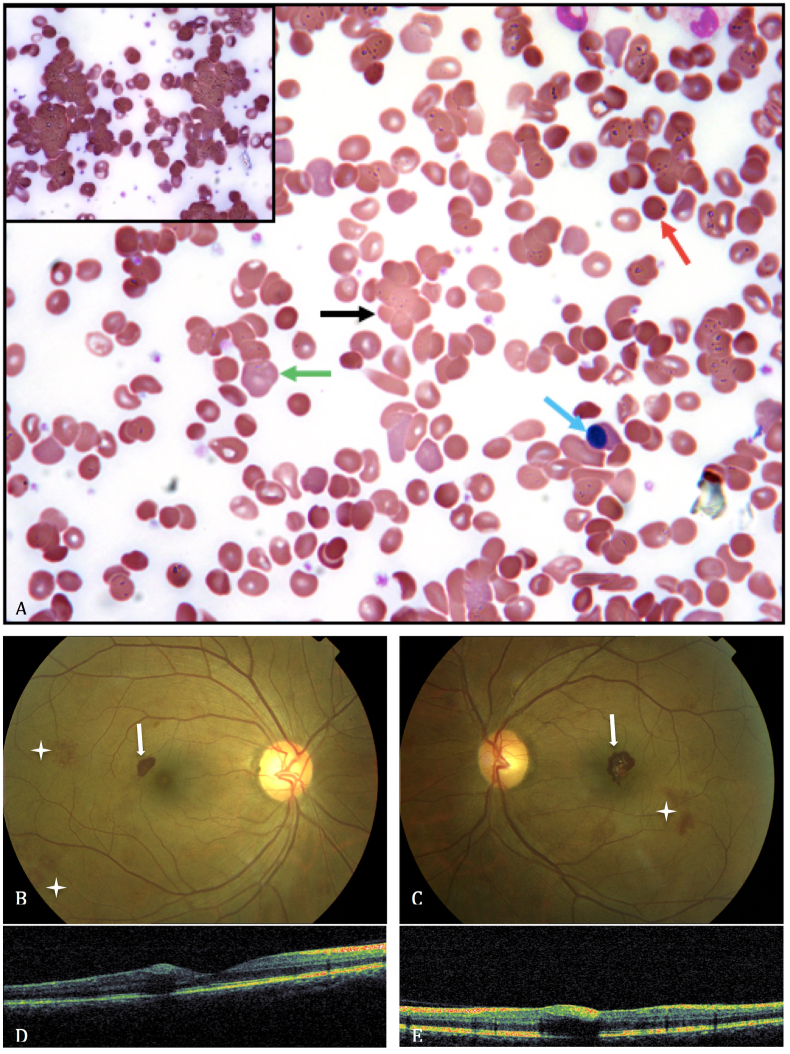
Legend Blood Smear, Retinography and Optical Coherence Tomography. **A**. Peripheral blood smear (May-Grünwald Giemsa, magnification: 400×). Erythrocyte agglutination is clearly detectable as clumping of red blood cells (black arrow, and upper left in detail); spherocytes can be recognized as round hyperchromic erythrocytes (red arrow); polychromasia is identifiable as larger, slightly bluer or purplish-stained red cells (green arrow) and indicates a robust regenerative response, as per hemolytic anemia; the presence of circulating orthochromatic erythroblasts (blue arrow) confirms the strong erythroid regenerative response. **B–C**. Retinography. Right parafoveal and left foveal hemorrhage (arrows) and mid-peripheral hemorrhages (stars). **D-E**. Optical Coherence Tomography. Hemorrhages beneath the internal limiting membrane with relative shadow cone, located in the perifovea in the right eye and obliterating the foveal depression in the left eye. (For interpretation of the references to colour in this figure legend, the reader is referred to the Web version of this article.)

**Fig. 2:**
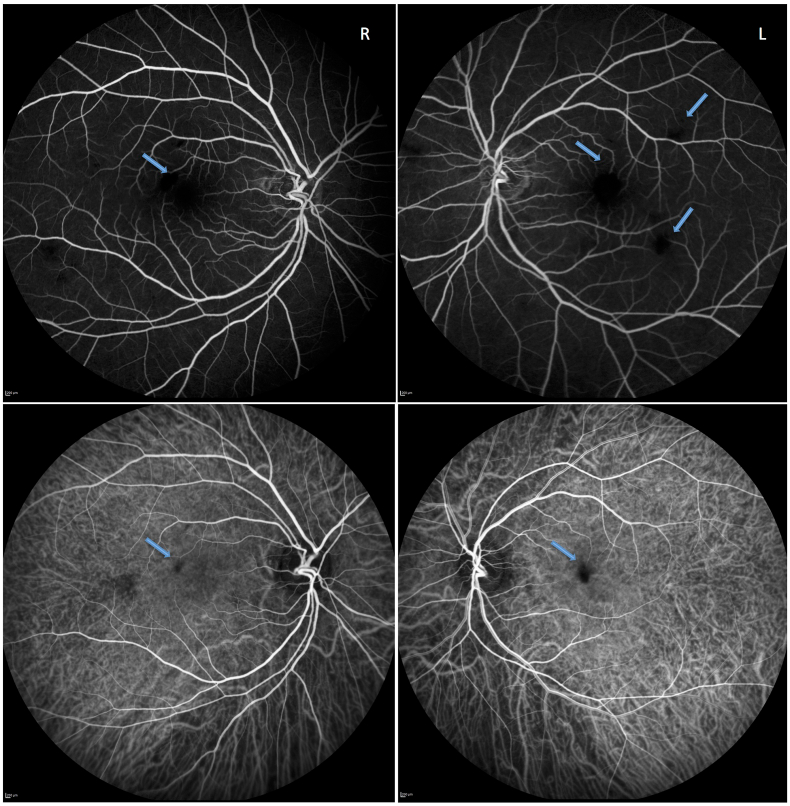
Fluorescein and Indocyanine Green Angiography. Images of the right (panel R) and left (panel L) eye. In the top row, the fluorescein angiography shows a bilateral blockage corresponding to the areas of hemorrhages (arrows). The retinal vascularization was otherwise unremarkable. In the bottom row, the indocyanine green angiography shows no involvement of the choroidal vascularization. Note the blockage due to the retinal hemorrhages (arrows). No signs of vasculitis, neovascularization or hypertensive retinopathy are present. (For interpretation of the references to colour in this figure legend, the reader is referred to the Web version of this article.)

On March 23, 2020, a 46-year-old North-African male was admitted to the Intensive Care Unit (ICU) of the University Hospital of Modena, Italy, for respiratory failure and unilateral visual loss after a 12-day history of fever, cough and worsening dyspnea. Medical history included well-controlled hypertension and hereditary spherocytosis with stable mild chronic hemolytic anemia (Hb mean values: 10–11 g/dL). Chest-X-Ray showed bilateral peripheral airspace opacities. SARS-CoV-2 infection was documented by reverse transcriptase-polymerase chain reaction on a nasopharyngeal swab. Bacterial co-infection was excluded. Blood tests showed severe anemia (Hb: 6.2 g/dL), with normal platelet and leukocytes counts, while levels of hemolytic markers were consistent with a hemolytic exacerbation (total Bilirubin (mostly indirect) was 1.8 mg/dl; Serum Lactate Dehydrogenase was 690 U/L (normal range 230–460); haptoglobin was 244 (normal range 34–200)). The peripheral blood smear (shown in Fig. 1-A) demonstrated the presence of several erythrocytes agglutinates (confirmed with direct antiglobulin test), along with spherocytes, polychromasia and circulating erythroblasts, so revealing mixed pathogenesis of the hemolytic process (cold-agglutinin-mediated and non-immune-mediated due to spherocytosis). G6PD levels were within normal limits. Concurrently, bilateral retinal hemorrhage with macular involvement was found. The patient was mechanically ventilated for 4 days, transfused with 3 units of packed red blood cells, and treated with Chloroquine. Progressive clinical improvement was observed, along with an increase of Hb up to 11.6 g/dL. The patient was discharged on the 6th April and referred for ophthalmic assessment. The visual acuity was 20/20 in the right eye and 20/70 in the left eye. Fundus examination showed few hemorrhages bilaterally, with right parafoveal and left foveal involvement (shown in Fig. 1-B, C). The latter was responsible for the central scotoma. Anterior segment, pupillary response and intraocular pressure were normal bilaterally. Findings on high-resolution optical coherence tomography demonstrated hemorrhages beneath the internal limiting membrane in both eyes, obliterating the foveal dip in the left one (shown in Fig. 1-D, E). Fluorescein and indocyanine green angiography showed bilateral blockage corresponding to the areas of hemorrhage in a context of normal circulation, excluding other causes of hemorrhages such as vascular occlusion, hypertensive retinopathy or vasculitis (shown in Fig. 2).

**Fig. 3:**
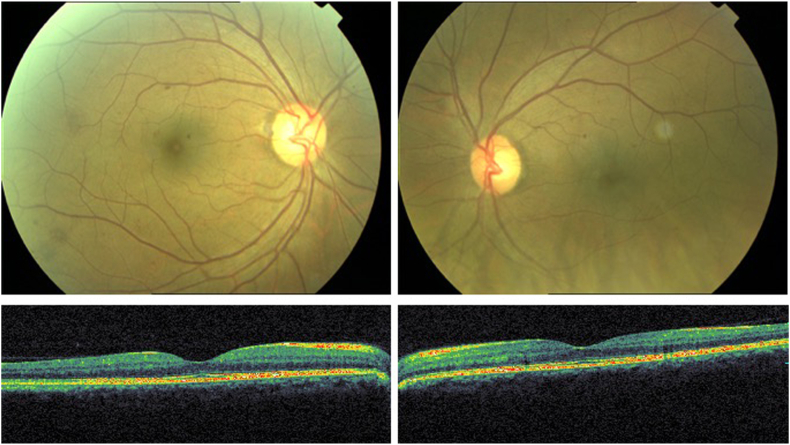
Retinography and Optical Coherence Tomography. Hemorrhages spontaneously improved at 1-month follow up.

No treatment was required and the hemorrhage spontaneously improved during 1 month, with consequent final visual acuity increase of 20/20 in both eyes (Fig. 3).

The patient has given his written informed consent to publish this case including publication of images.

## 3 Discussion/conclusion

The co-occurrence of severe COVID-19 symptoms and visual loss suggested a causal link. Recently, a case series reported retinal findings in patients with COVID-19, however in this series, the presence of differentials underlying conditions was not investigated as potential cause of retinal damage.^2^

The prevalence of retinopathy in patients with severe anemia is 28%^3^ and a pathogenesis based on autoimmune hemolytic anemia (AIHA) was anecdotally reported in few cases.^4^^,^^5^

In AIHA genetic mutations in plasma membrane proteins lead to a greater risk for hemolysis process due to the instability of red blood cell membrane-cytoskeleton interaction. Some particular conditions have been considered as triggers of hemolysis including fever, viral infection or hypoxia.^6^ In the hemolytic anemia condition, the exact mechanism of retinal damage is not entirely understood, although hypoxia seems to be a critical factor. The compromised erythrocyte deformability would be responsible of an increased blood viscosity and prothrombotic state with a consequent retinal non-perfusion.^6^ The latter can lead to vascular enlargement, increased transmural pressure and microtraumas to the vessel walls, which can cause retinal cotton wool spots, edema and bleedings. We hypothesize that SARS-CoV-2 may have played a role in the onset of an acute, transient cold-agglutinin hemolytic anemia, on a backdrop of chronic mild anemia, thus eventually eliciting the retinal bleeding. Cold agglutinins have been, indeed, described in association with different infectious agents as a result of phenomena of cross-reactivity, and SARS-CoV-2 seems strikingly able to induce dysregulated immune responses^7^; furthermore, in our patient, it is conceivable that the underlying membrane defect may have yielded the red cells more prone to the hemolytic process. Since ocular manifestations appear to correlate with the severity of COVID-19,^8,9^ophthalmologists should be aware of uncommon ophthalmic consequences, following the multifaceted systemic (often immune-mediated) effects of SARS-CoV-2 infection.

## Patient consent

Written consent to publish this case report has been obtained from the patient. This report does not contain any personal identifying information.

## Funding

No funding or grant support.

## Authorship

All authors attest that they meet the current ICMJE criteria for Authorship.

## Data availability statement

All data are available on a reasonable request to the corresponding author.

## Author contributions

Rodolfo Mastropasqua, Vincenzo Nasillo and Rossella D'Aloisio wrote and revised the manuscript; Matteo Gironi collected data and performed the clinical evaluation. All of the authors approved the final version of the manuscript for publication.

## Declaration of competing interest

The authors declare that they have no known competing financial interests or personal relationships that could have appeared to influence the work reported in this paper.

## References

[1] van der Hoek L., Pyrc K., Jebbink M.F. (2004). Identification of a new human coronavirus. Nat Med.

[2] Marinho P.M., Marcos A.A.A., Romano A.C., Nascimento H., Belfort R. (2020). Retinal findings in patients with COVID-19. Lancet.

[3] Carraro M.C., Rossetti L., Gerli G.C. (2001). Prevalence of retinopathy in patients with anemia or thrombocytopenia. Eur J Haematol.

[4] Oner A., Ozkiris A., Dogan H., Erkilic K., Karakukcu M. (2005). Bilateral macular hemorrhage associated with autoimmune hemolytic anemia. Retina.

[5] Thomas A.S., Walter S.D., Fekrat S. (2016). Bilateral prefoveal sub-internal limiting membrane hemorrhage in autoimmune hemolytic anemia. Ophthalmic Surg Lasers Imaging Retina.

[6] Huggins A.B., Garg S.J., JrRS Sando (2015). Central retinal vein occlusion in hereditary spherocytosis. Retin Cases Brief Rep.

[7] Chen L., Liu M., Zhang Z. (2020). Ocular manifestations of a hospitalised patient with confirmed 2019 novel coronavirus disease. Br J Ophthalmol.

[8] Wu P., Duan F., Luo C. (2020). Characteristics of ocular findings of patients with coronavirus disease 2019 (COVID-19) in hubei province, China. JAMA Ophthalmol.

